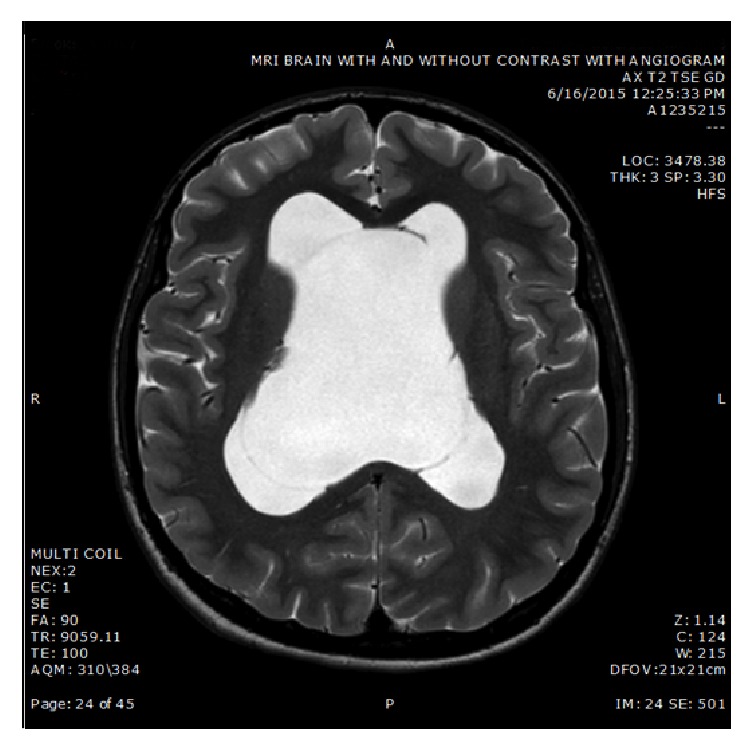# Levodopa-Responsive Early-Onset Parkinsonism in Down Syndrome

**DOI:** 10.1155/2018/2314791

**Published:** 2018-02-15

**Authors:** Padmini Palat, Francis Hickey, Lina Patel, Elise Sannar

**Affiliations:** Children's Hospital Colorado, 13123 E. 16th Ave., Aurora, CO 80045, USA

## Abstract

Individuals with Down syndrome (DS) can develop Alzheimer's disease as early as 30 to 40 years old, but parkinsonism is rarely described. We report on a 20-year-old woman with Down syndrome and parkinsonism who responded dramatically to carbidopa-levodopa. We propose that the occurrence of parkinsonism in individuals with DS may be underreported. Recognizing and treating this condition may improve quality of life.

## 1. Case Report

AB, a 20-year-old woman with Down syndrome (DS) and moderate intellectual disability, was referred to a Down syndrome clinic for loss of skills. Her medical history was significant for atrial septal defect repair in infancy and Graves' disease in remission for the last four years off medications. She presented with a one-year history of slowing in her general movements and with behavioral regression. The family noted that her symptoms became more noticeable when she changed day programs from a less structured to a more demanding environment. The change was implemented because she had been so successful the prior year. She previously spoke in full sentences, but now her speech was soft and limited to 1 or 2 words at a time. She had tremors that had increased significantly over a few months. These motor symptoms impacted her ability to complete activities of daily living. She had occasional bowel or bladder accidents if she was not able to get to a bathroom in time. The patient exhibited some symptoms of depression, including decreased interest in activities and somber affect. The decline in function did not appear to affect her comprehension as she was still able to follow conversations and directions as before, albeit more slowly. Neurological findings were significant for paucity of expressive speech, hypophonia, decreased facial expression, slow gait, tremors, and cogwheel rigidity. Her motor findings were more pronounced on the left side.

Extensive testing including complete blood count, celiac panel, thyroid panel, vitamin B12 level, HIV screen, complete metabolic panel, sedimentation rate, urine HVA/VMA, ANA, paraneoplastic panel, and myasthenia gravis antibodies was unremarkable. Her EEG showed mild diffuse slowing consistent with her diagnosis of DS. An MRI of her brain showed cystic expansion of the cavum septum pellucidum and vergae, measuring 7.5 cm in greatest transverse dimension. The lateral ventricles were enlarged and obstructed at the level of the foramina of Monro with no obvious periventricular interstitial edema (See Figure 1). Follow-up images three months, one year, and two years later remained stable. A CT scan of her brain did not show calcifications.

Developmental regression related to depression was initially suspected, leading to mental health interventions. Her psychiatrist started citalopram, which improved her affect and level of interest, but not her motor slowing. The neurologist initiated carbidopa-levodopa 25 mg–100 mg three times daily for symptomatic treatment of extrapyramidal features. This led to an immediate and dramatic response with return to baseline motor function. The patient had no side effects of treatment and sustained the response for a fairly long period on the same dose of carbidopa-levodopa. Follow-up examination eighteen months after initiation of therapy showed recurrence of cogwheel rigidity and increase in tremors, requiring doubling of the dose of carbidopa-levodopa and giving it four times a day. She responded well to the dose increase and has remained functionally stable for more than two years without cognitive decline.

## 2. Discussion

Down syndrome (DS) is the most frequently occurring chromosomal disorder and the leading cause of intellectual disability in the United States. The incidence of DS is about 1/700 live births. The life expectancy of individuals with DS is currently in the 60s with adequate healthcare and social support. Alzheimer's disease (AD) occurs much earlier in individuals with DS, who can develop dementia starting in the 30s.

Parkinson's disease and parkinsonism occur due to deficiency of the neurotransmitter dopamine in the substantia nigra. The drug levodopa is the most important first-line drug for the management of Parkinson's and is converted to dopamine in the brain. It is almost always given in combination with carbidopa, which is a dopamine enhancer and prevents the nausea that can be caused by levodopa alone.

There are some reports in the literature describing parkinsonian features in individuals with DS and Alzheimer's. In a study of 53 individuals with DS and dementia, Lai and Williams found that up to 20% had extrapyramidal signs [1]. Brain CT scans showed tissue loss, particularly in the temporal lobes. Two patients who presented with parkinsonism showed signs of dementia half a year later. Five out of 10 patients who were prescribed carbidopa-levodopa responded to treatment. Vieregge et al. reported extrapyramidal features in 36% of patients with DS and AD (*n* = 54; mean age 44) [2]. Brandel et al. described a 45-year-old man with DS and parkinsonism who responded to levodopa but also had loss of initiative, concentration, and memory [3]. In contrast, there is only one previous case report of parkinsonism without cognitive decline in DS. Singer et al. described a 43-year old man with DS and parkinsonism who had a normal head CT, responded to levodopa, and had no mental decline over two years [4].

Pathological studies have shown decreased dopamine content in the basal ganglia in DS with AD. Senile plaques and neurofibrillary tangles sufficient to make a neuropathological diagnosis of AD are well established in middle-aged individuals with DS but there are conflicting reports as to whether Lewy bodies occur more frequently in age-matched brains of patients with DS compared to controls [5]. Raghavan et al. performed a clinicopathological study of 23 individuals with DS aged 49 to 74 [6]. Two patients aged 50 and 56 were found to have Lewy body formation in the substantia nigra in addition to cortical Alzheimer-like pathology. The authors postulated a correlation with DS and Parkinson's disease.

Hydrocephalus is rare in DS and cavum septum pellucidum cyst has been reported prenatally in a single case of DS [7]. Parkinsonian features can occur in hydrocephalus due to involvement of terminal dopaminergic neurons bordering the lateral ventricles [8] or due to mechanical distortion and consequent vascular insufficiency in the nigrostriatal system. However, these cases respond to ventricular shunting rather than to levodopa.

Basal ganglia calcifications are described in DS and are generally attributed to early aging of the brain [2]. However, no specific reports exist of associated parkinsonian symptoms.

Parkinson's disease also has a known association with depression, implicating other neurotransmitter systems including serotonin and norepinephrine. Treatment with the dopamine precursor levodopa addresses motor dysfunction but does not necessarily improve depressive symptoms. Some cases of depression in Parkinson's are not responsive to SSRIs, implicating a potentially distinct pathophysiology from major depressive disorder [9].

Finally, motor manifestations including symptoms of catatonia can be seen in severe cases of regression in DS [10]. This phenomenon is reported in adolescence or young adulthood, often associated with major life transitions. Some cases of regression in DS are responsive to antidepressant therapy.

## 3. Conclusion

This young woman with Down syndrome (DS) presented with classical features of parkinsonism. She has maintained her response to levodopa for two years.

Individuals with DS can get neurological complications from strokes, autoimmune disorders, moyamoya syndrome, endocrine dysfunction, epilepsy, or cervical cord compression, but these were excluded in our patient by appropriate testing. Our patient had a ventricular cyst that was initially concerning for normal pressure hydrocephalus (NPH) but she lacked other features of NPH such as bladder incontinence and the characteristic gait abnormality. In addition, she showed stable brain imaging over two years and responded dramatically to levodopa, making NPH unlikely. Her clinical findings preceded treatment with antidepressant medication and do not seem medication-related. Familial PD usually occurs at a later age, though a family history was not available as the patient was adopted. It is debatable whether her citalopram-responsive depression was a manifestation of parkinsonism or a separate disease process. Regression in behavior triggered by a lifestyle change may also have responded to the SSRI, but her improvement with levodopa suggests the parkinsonism was an independent entity.

Our patient is unique due to her young age of onset of parkinsonism, her dramatic and sustained response to levodopa, and the absence of cognitive decline. Individuals with DS who present with extrapyramidal symptoms may be mistaken to have psychomotor slowing due to Alzheimer's; therefore, the occurrence of parkinsonism may be underreported. A multidisciplinary approach is useful in recognizing associated comorbidities such as depression. We recommend a trial of levodopa if parkinsonian features are observed on neurological examination in an individual with DS.

## Figures and Tables

**Figure 1:**